# Optimizing β‐lactam‐containing antibiotic combination therapy for the treatment of Buruli ulcer

**DOI:** 10.1111/bcp.16209

**Published:** 2024-09-18

**Authors:** Salvatore D'Agate, Peter Velickovic, Noelia García‐Barrios, Santiago Ramón‐García, Oscar Della Pasqua

**Affiliations:** ^1^ Clinical Pharmacology & Therapeutics Group University College London London UK; ^2^ Research and Development Agency of Aragón Foundation (ARAID Foundation) Zaragoza Spain; ^3^ Department of Microbiology, Faculty of Medicine University of Zaragoza Zaragoza Spain; ^4^ Spanish Network for Research on Respiratory Diseases (CIBERES), Carlos III Health Institute Health Institute Carlos III Madrid Spain; ^5^ National Research Council (CNR) Rome Italy

**Keywords:** amoxicillin/clavulanic acid, Buruli ulcer, clinical trial simulations, dose rationale, pharmacokinetic–pharmacodynamic modelling

## Abstract

**Aims:**

The current treatment for Buruli ulcer is based on empirical evidence of efficacy. However, there is an opportunity for shortening its duration and improving response rates. Evolving understanding of the pharmacokinetic–pharmacodynamic relationships provides the basis for a stronger dose rationale for antibiotics. In conjunction with modelling and simulation, it is possible to identify dosing regimens with the highest probability of target attainment (PTA). This investigation aims to: (i) assess the dose rationale for a new combination therapy including amoxicillin/clavulanic acid (AMX/CLV) currently in clinical trials; and (ii) compare its performance with alternative dosing regimens including rifampicin, clarithromycin and AMX/CLV.

**Methods:**

In vitro estimates of the minimum inhibitory (MIC) concentration were selected as a measure of the antibacterial activity of different drug combinations. Clinical trial simulations were used to characterize the concentration *vs*. time profiles of rifampicin, clarithromycin and amoxicillin in a virtual cohort of adult and paediatric patients, considering the effect of baseline covariates on disposition parameters and interindividual variability in exposure. The PTA of each regimen was then assessed using different thresholds of the time above MIC.

**Results:**

A weight‐banded dosing regimen including 150–600 mg rifampicin once daily, 250–1000 mg clarithromycin and AMX/CLV 22.5 mg/kg /1000 mg twice daily ensures higher PTA than the standard of care with AMX/CLV 45 mg/kg/2000 mg once daily.

**Conclusion:**

The higher PTA values support the proposed 4‐drug combination (rifampicin, clarithromycin, AMX/CLV) currently under clinical investigation. Our findings also suggest that higher rifampicin doses might contribute to enhanced treatment efficacy.

**What is already known about this subject**

- Buruli ulcer is a neglected tropical skin disease that mainly affects poor communities in remote rural areas of West Africa.
- Recently, promising results have been observed in an in vitro experimental protocol in which amoxicillin/clavulanic acid (AMX/CLV) were tested in combination with rifampicin and clarithromycin.
- Pharmacokinetic–pharmacodynamic principles can be used to predict the antibacterial activity of novel regimens and antibiotic drug combinations in humans.

**What this study adds**

- The use of pharmacokinetic modelling and simulation in conjunction estimates of the probability of target attainment enabled the identification of suitable regimens including AMX/CLV for further evaluation in efficacy trials.
- Adding currently approved doses of AMX/CLV (i.e., AMX/CLV 22.5 mg/kg/1000 mg twice daily) to standard of care yields higher antibacterial activity against different strains of *Mycobacterium ulcerans*.
- Our findings also suggest that higher doses of rifampicin may contribute to enhanced treatment efficacy.

## 1 INTRODUCTION

Buruli ulcer (BU), also called *Mycobacterium ulcerans* disease, is a chronic infectious disease that primarily affects the skin and soft tissue. Without treatment, it frequently progresses to massive ulceration and bone damage. Although mortality is low, permanent disfigurement and disability are high, occurring in up to 25% of the cases and stigmatizing the affected populations. BU is a neglected tropical skin disease that mainly affects poor communities in remote rural areas of West Africa with limited access to health care, although it is also present in other parts of the world.^1^, ^2^ There are no effective preventative measures in humans. Although its mode of transmission has been elucidated in Australia, it is still unknown in the African context.^3^

Before 2004, surgical intervention through excision followed by skin grafting was the only option available as treatment of BU. At that time, antibiotic‐based BU therapy was introduced and it has been increasingly used.^4^ Current World Health Organization (WHO) guidelines recommend a treatment consisting in a combination of two oral antibiotics over the course of 8 weeks for all forms of active disease, namely rifampicin (RIF) at 10 mg/kg body weight daily and clarithromycin (CLA) at 7.5 mg/kg body weight twice daily.^2^, ^4^ [Corrections made on 24 September 2024, after first online publication: In the preceding sentence, the link to rifampicin and clarithromycin has been corrected in this version.] However, the requirement for an 8‐week treatment has also proven to be problematic, as adherence to therapy is variable due to difficulties in the access to medicines and the need in some cases of prolonged hospitalizations, which impacts household's economies.^5^ Similarly to ongoing efforts in the battle against tuberculosis, it is envisaged that addition of another component to the current combination therapy may enable treatment shortening, which will simplify the control of the disease.

Beta‐lactams are one of the largest groups of antibiotics available today with an exceptional record of clinical safety both in adults and children.^5^ Recently, promising results have been observed in an in vitro experimental protocol in which amoxicillin (AMX)/clavulanic acid (CLV) were tested in combination with RIF and CLA.^6^ [Corrections made on 24 September 2024, after first online publication: In the preceding sentence, the link to amoxicillin has been corrected in this version.] Fractional inhibitory concentration index estimates showed that the quadruple combination results in a synergistic interaction, as opposed to *no interaction* associated with the two drugs used as standard of care. These findings offer an opportunity to explore alternative regimens in the clinic and were the basis of two currently ongoing clinical trials in West Africa.^7^ The dose rationale for these assays was selected based on pragmatic approximations in the field and with the goal of subsequent WHO endorsement to facilitate implementation by the National Control programmes. To this aim, the WHO recommended backbone treatment (RIF + CLA) was maintained and AMX/CLV, which is typically dosed thrice daily, was chosen to be administered at the highest recommended dose only twice daily to match that of CLA dosing to ensure patient compliance, while maintaining once daily RIF. In addition, a generic formulation of AMX/CLV was used in order to swiftly ensure access to medicines and facilitate country implementation.

The aims of this study were therefore: (i) to confirm the pragmatic regimen that was selected for the current clinically tested combination of AMX/CLV^7^; and (ii) to identify a new, potentially improved, posology for the combination therapy of RIF/CLA including AMX/CLV. Our investigation provides the basis for the optimisation of a prospective clinical trial in BU patients taking into account the effect of interindividual variability in pharmacokinetics (PK) as well as bacterial susceptibility based on data from different clinical isolates of *M. ulcerans*.

Using a model‐based approach in conjunction with clinical trial simulations,^8^, ^9^, ^10^ we attempt to predict the overall performance of 9 different regimens including AMX/CLV in a virtual cohort of adult and paediatric patients. The goal is to maximize exposure to AMX/CLV, so that the optimal ratio between individual components can be identified for the development and evaluation of fixed‐dose combination regimens.

## 2 METHODS

Clinical trial simulations for the evaluation of the overall performance of AMX/CLV‐containing regimens were implemented under the assumption that the observed increase in antibacterial activity of the combination is associated with the time‐dependent mechanism(s) by which β‐lactams act, i.e., based on the fraction or proportion of the time that concentrations remain above the minimum inhibitory concentration (MIC). The probability of target attainment (PTA) was based on the AMX concentration *vs*. time profiles that remain at least 40% of the time above the MIC (T > MIC). In this approach, the contribution of the other drugs to the antibacterial activity of the combination is parameterised in terms of the changes in MIC.

**FIGURE 1:**
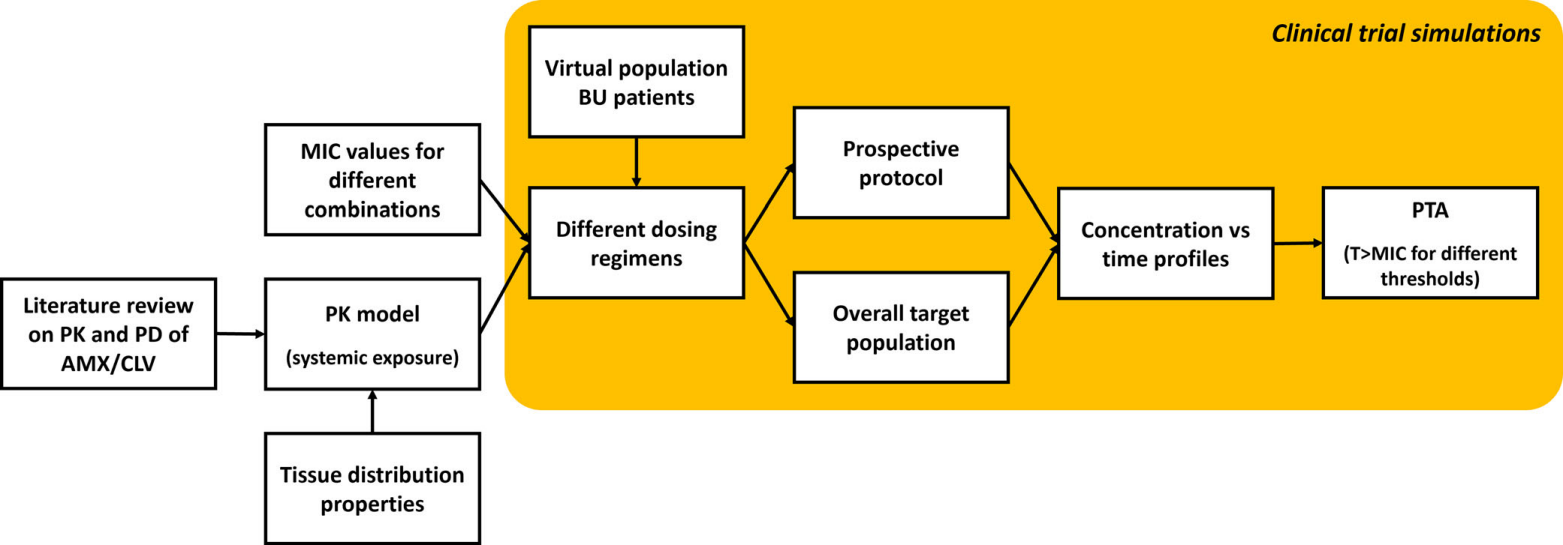
Workflow and steps required for the implementation of the clinical trial simulations. In addition to exploring different doses and dosing regimens for each of the individual components in the combination, simulations scenarios included a virtual population likely to reflect the target (African) population to be enrolled in a prospective protocol, taking into account the incidence of Buruli ulcer (BU) by age. AMX/CLV, amoxicillin/clavulanic acid; MIC, minimum inhibitory concentration; PD, pharmacodynamic; PK, pharmacokinetic; PTA, probability of target attainment.

An outline of the steps undertaken to ensure realistic estimates of PTA is shown in Figure 1. A preliminary review of the data on the prevalence and incidence of BU by age group was performed along with an evaluation of the available PK models describing the absorption and disposition of different dosage forms of combinations containing AMX and CLV.

### 2.1 In vitro MICs and clinical isolates

We have used previously reported data from the NCTC 10417 strain and 5 clinical isolates (namely, ITM 06384, ITM C05142, ITM C05143, ITM C08756 and ITM M000932).^6^ An overview of the MIC values for the lab strain and clinical isolates included in the current analysis is summarized in Table 1. For comparison, estimates of the MIC of the RIF + CLA combination were 0.025 and 0.125 (μg/mL).^6^ It should be noted that based on the fractional inhibitory concentration index, the combined use of RIF and CLA does not result in an interaction, as the fractional inhibitory concentration index is 0.75 (i.e., no interaction).

**TABLE 1:** Minimum inhibitory concentration (MIC) values used as threshold for bacterial susceptibility and subsequent calculation of the probability of target attainment. Full details of the concentrations used in the in vitro protocol can be found in Arenaz‐Callao et al.^6^. The reported in vitro MIC values correspond to the lowest possible concentration of every compound that, when assayed together, prevented bacterial growth. Consequently, different MIC values were obtained depending on whether amoxicillin was tested alone or in combination.

Strain	AMX/CLV + RIF + CLA (μg/mL)	AMX/CLV + RIF (μg/mL)	AMX/CLV + CLA (μg/mL)	AMX/CLV (μg/mL)
NCTC 10417 (ATCC19423)	0.063	0.06	2	4
ITM 06384	1	0.125	4	0.5
ITM C05142	0.0625	0.125	0.125	0.5
ITM C05143	4	2	2	8
ITM C08756	0.25	0.125	0.25	0.5
ITM M000932	0.125	0.125	2	2

Abbreviations: AMX/CLV, amoxicillin/clavulanic acid; CLA, clarithromycin; RIF, rifampicin.

### 2.2 PK models

To simulate concentration *vs*. time profiles for each drug in the combination we implemented PK models based on previously published data. For RIF the model described by Svensson *et al.* ^11^ was used, while for CLA, the model by Abduljalil *et al.* ^12^ was chosen. As significant differences in parameterisation were observed for AMX in the adult and paediatric population, two models were used to ensure accurate description of the individual profiles across the age range of interest. For adults and adolescents aged ≥15 years, the model developed by Carlier *et al.* ^13^ was adapted, including absorption and bioavailability estimates from a study by de Velde *et al.* ^14^ For the paediatric population aged 5–14 years, individual profiles were generated using the model developed by De Cock *et al.* ^15^ In contrast to the other models, De Cock *et al.* used cystatin C and postmenstrual age to describe the effect of age and maturation processes on clearance, which were relevant for the population included in their original publication. Additionally, unlike creatinine, cystatin C predicts glomerular filtration rate in children independent of age, sex, height and body composition.^16^ The PK of CLV was not simulated, as its levels were considered pharmacologically active throughout the course of treatment, irrespective of interindividual variation in exposure. The absorption rate constant of AMX in both models was set to 0.66 h^−1^.^17^

Individual concentrations were derived based on a blood sampling scheme including samples every 15 min over the period of 28 days, to ensure that steady state conditions were achieved for all components of the combination and account for the effect of RIF autoinduction. These profiles were assumed not to be time variant, and as such reflect drug exposure after a 4‐ and 8‐week treatment. Random and specific sources of variation in PK were included and therefore stochastic (i.e., interindividual variability) and deterministic (i.e., baseline clinical and demographic covariate factors) effects were considered.

### 2.3 Clinical trial simulations

Simulation scenarios were implemented according to a parallel study protocol design including adult and paediatric patients. PK parameter estimates obtained previously were used to simulate individual PK profiles of AMX following oral administration of different doses and dosing regimens. A stepwise outline of the simulation procedures is provided in the next paragraphs.

#### 2.3.1 Virtual patient cohorts

Initially, a virtual cohort consisting of 140 paediatric and adult patients (cohort 1) was generated to mimic the study population to be recruited in two prospective clinical studies in Benin, Côte d'Ivoire, Ghana and Togo (the *Beta‐Lactam Containing Regimen for the Shortening of Buruli Ulcer Disease Therapy* study [ClinicalTrials.gov Identifier: NCT05169554] and the *Shortening Buruli Ulcer treatment: WHO recommended vs. a novel β‐lactam‐containing therapy – Phase III evaluation in West Africa* study [Pan African Clinical Trials Registry Identifier: PACTR202209521256638])(the BLMs4BU Consortium). These studies aim to recruit patients between the ages of 5 and 70 years, with a body mass index of <30 kg/m^2^ and normal renal function, as assessed by creatinine clearance within the range from 50 to 150 mL/min, excluding subjects with hyperfiltration or renal impairment.^18^ To reflect demographic data of populations in West Africa, where the ongoing clinical trials are being conducted, data for growth parameters of the paediatric population (including height, weight and body mass index) was obtained from a previously conducted cross sectional study of children, aged 6–18 years in Calaba, Nigeria.^19^ Growth parameters for children from the study are listed in Table S1. To accurately reflect the age distribution at enrolment, reported data by the WHO on the prevalence and incidence of BU in children and adults were used.^2^, ^20^, ^21^ Accordingly, incidence of BU was assumed to be 48% in the paediatric population (<15 years old) and 52% among adolescents >15 years old and adults.

Subsequently, a second cohort (cohort 2) based on the population available in the NHANES database, was created taking into account the West African paediatric growth parameters to better characterize the effect of clinical and demographic covariate on the disposition of AMX. This cohort consisted of 100 subjects per weight group, stratified into 9 weight brackets from 11 to 100 kg. Brackets were created with intervals of 10 kg: 11 to <20, 20 to <30, 30 to <40, 40 to <50, 50 to <60, 60 to <70, 70 to <80, 80 to <90, 90 to <100 kg. In this cohort, age, weight and renal function are assumed to be determinants of the interindividual variability of clearance and volume of distribution. The implications of variability due to differences in formulation are not factored into the current analysis. It is hypothesised that formulations are approved according to international regulatory standards for bioequivalence.

#### 2.3.2 Simulation scenarios

Individual concentration *vs*. time profiles were generated for cohorts 1 and 2 using the baseline characteristics identified as covariates and parameter estimates from the PK models described above. The doses and dosing regimens included in the different simulation scenarios are summarized in Table 2.

**TABLE 2:** Simulation scenarios for the evaluation of the probability of target attainment of β‐lactam containing regimens for the treatment of paediatric and adult patients with BU. All drugs included in the scenarios were administered according to once daily (q.d.) or twice daily (b.i.d.) weight‐banded regimens. Four different dose levels were evaluated for rifampicin (RIF) and clarithromycin (CLA), and 2 dose levels for amoxicillin/clavulanic acid (AMX/CLV).

Scenario	Doses and dosing regimens	Note
*0*	RIF 150/300/450/600 mg q.d. + CLA 250/500/750/1000 mg b.i.d.	Reference standard of care. This scenario was only used for graphical summaries
*1*	RIF 150/300/450/600 mg q.d. + CLA 250/500/750/1000 mg b.i.d. + AMX/CLV 22.5 mg/kg or 1000 mg b.i.d.	Regimen proposed for study NCT05169554 (See Table S2 for details)
*2*	RIF 150/300/450/600 mg q.d. + CLA 250/500/750/1000 mg b.i.d. + AMX/CLV 45 mg/kg or 2000 mg q.d.	Standard of care in combination with 45 mg/kg or 2000 mg AMX/CLV q.d.
*3*	RIF 75/150/225/300 mg b.i.d. + CLA 250/500/750/1000 mg b.i.d. + AMX/CLV 22.5 mg/kg or 1000 mg b.i.d.	RIF dose is maintained but is administered as a b.i.d. regimen
*4*	RIF 150/300/450/600 mg b.i.d. + CLA 250/500/750/1000 mg b.i.d. + AMX/CLV 22.5 mg/kg or 1000 mg b.i.d.	RIF dose is doubled and administered as a b.i.d. regimen
*5*	RIF 150/300/450/600 mg q.d. + AMX/CLV 22.5 mg/kg or 1000 mg b.i.d.	Combination does not include CLA
*6*	RIF 300/600/900/1200 mg q.d. + CLA 250/500/750/1000 mg b.i.d. + AMX/CLV 22.5 mg/kg or 1000 mg b.i.d.	Regimen proposed for study PACTR202011867644311 (high RIF) plus AMX/CLV. Treatment linked to dialkylcarbamoyl chloride‐coated dressings
*7*	RIF 525/1050/1575/2100 mg q.d. + CLA 250/500/750/1000 mg b.i.d. + AMX/CLV 22.5 mg/kg or 1000 mg b.i.d.	Increased RIF dose in combination with CLA and AMX/CLV
*8*	CLA 250/500/750/1000 mg b.i.d. + AMX/CLV 22.5 mg/kg or 1000 mg b.i.d.	Combination does not include RIF

*Note*: Weight bands.

1. RIF dose 1 (11 ≤ x ≤ 20), dose 2 (21 ≤ x < 40 kg), dose 3 (40 ≤ x ≤ 54 kg) or dose 4 (>54 kg).

2. CLA dose 1 (11 ≤ x ≤ 20 kg), dose 2 (21 ≤ x < 40 kg), dose 3 (40 ≤ x ≤ 54 kg) or dose 4 (>54 kg).

3. AMX/CLV dose 1 (≤40 kg), dose 2 (>40 kg).

Moreover, the effect of skin distribution of AMX was assessed for cohort 1. Following a literature search, studies were identified, which detail the characterization of the free (unbound) concentration of all 3 drugs and skin exposure to AMX.^22^, ^23^, ^24^ The predicted PTA based on *M*. *ulcerans* strain NCTC 10417 (ATCC19423) was calculated considering the ratio of the area under the concentration *vs*. time curve (AUC) in skin tissue to the AUC of plasma derived from this study (0.163).

Additionally, the potential drug–drug interaction between RIF and CLA was included in the simulated scenarios.^25^ It has been shown that RIF‐mediated induction of CYP450 isoenzymes leads to significant decrease in the concentrations of CLA (i.e., up to 67%). As the 14‐hydroxy‐CLA (M‐5) metabolite shows very limited antibacterial activity, changes in metabolite formation were not considered clinically relevant.

Using the MIC values for AMX/CLV reported in Table 1, T > MIC, was calculated for each individual profiles under the assumption of minor formulation‐related variation. Over the course of the entire treatment duration, the MIC value was considered to vary depending on whether the concentration of the other antibiotics in the combination were below or above those that were used experimentally to determine the antibacterial activity of the combinations. For instance, if the concentration of RIF was below its own MIC value, the instantaneous MIC value for AMX/CLV was the one observed without RIF. If both RIF and CLA were below their own MIC values, AMX/CLV was considered as acting alone.

Different thresholds for T > MIC were considered for the proposed doses and dosing regimens, namely 40, 50, 60, 75, 85, 90 and 95%. In vitro and in vivo animal studies suggest that efficacy is achieved for Gram‐positive bacteria when the percentage T > MIC of the unbound serum concentration is >40%.^26^ The assessment of different thresholds was useful for the interpretation of the results and potential clinical relevance of the differences observed between doses and dosing regimens for the PTA.

It is worth noting that the PTA is a complementary metric that encompasses not only the effect of pharmacodynamic variability (i.e., MIC differences) but also that of inter‐ and intraindividual variation in exposure due to both PK and pharmaceutical (i.e., formulation) factors. It is obtained by calculating the proportion of subjects above the target T>MIC. Comparison of doses and dosing regimens based on the PTA offers an opportunity to assess the overall treatment performance of each combination considering the different sources of variability. As the primary threshold of interest was T_40%_ > MIC, the PTA indicates the proportion of subjects whose concentration *vs*. time profile results in levels that remain at least 40% of the time above the relevant MIC for the combination. Recommendations were to be made based on dose(s) and dosing regimen(s) that resulted in PTA values >90%.

Secondary PK parameters were also derived and summarized (medians, 5^th^ and 95^th^ percentiles), including AUC_0‐24_ describing the systemic exposure over a 24 h period, Cmax_ss_, the maximum concentration at steady state and Cavg_ss_, the average steady‐state concentration. When appropriate, data were stratified by age or body weight.

To account for random (unexplained variability), each simulation scenario was replicated 100 times for cohort 1. For the sake of clarity, PTA results are summarized per cohort as a typical clinical trial setting, i.e. graphical summaries describe the results from a single simulation (iteration) with a cohort of n = 140, whereas numerical summaries reflect the results from 100 simulations (n = 140 x 100), including the 90% prediction intervals for the PTA. All data handling, including preparation, and subsequent creation of statistical and graphical summaries was performed in R using Rstudio (version 1.4.1106).^27^ Simulation scenarios were evaluated in NONMEM 7.5.1^28^ (ICON Development Solutions, Maryland, USA), supported by PsN (5.0.0) (University of Uppsala, Sweden). [Corrections made on 23 September 2024, after first online publication: In the preceding sentence, 7.5I has been changed to 7.51 in this version.] PIRANA Version 2.9.9 (Pirana‐Software, The Netherlands) was used for model management, execution and output generation. No formal statistical hypothesis testing was used to assess the statistical significance level of differences between scenarios.

#### 2.3.3 Nomenclature of targets and ligands

Key protein targets and ligands in this article are hyperlinked to corresponding entries in http://www.guidetopharmacology.org/, and are permanently archived in the Concise Guide to PHARMACOLOGY 2023/24.^29^

## 3 RESULTS

Baseline demographic characteristics of the patient population included in the simulation scenarios are summarized in Table 3. Accounting for the disease incidence by age group, Cohort 1 consisted of 67 patients aged from 5 to 15 years old and 73 aged >15 years old.

**TABLE 3:** Summary of the demographic characteristics of the virtual cohorts used in the clinical trial simulations. See text for details on cohorts 1 and 2. Data are presented as a median (range).

Baseline characteristics	Cohort 1 (*n* = 140)	Cohort 2 (*n* = 900)
Age (years)	16 (5–70)	18 (5–70)
Children <12 years:*	36 (25.5)	265 (29.5)
Adolescents 12–17 years:*	47 (33.5)	176 (19.5)
Adults ≥18 years:*	57 (41)	459 (51)
Height (cm)	157.1 (109.8–188.6)	159.2 (107.9–196.6)
Weight (kg)	52.15 (17.7–88.2)	55.6 (15.9–99.6)
Body mass index (kg/m^2^)	20.90 (12.48–29.9)	21.4 (11.5–29.9)
Creatinine clearance (mL/min)	97.6 (51.7–149.7)	95.7 (50.1–149.5)
Cystatin C (mg/L)	0.865	0.865

* Values between parentheses represent the percentage (%) of subjects relative to the total cohort population.

**FIGURE 2:**
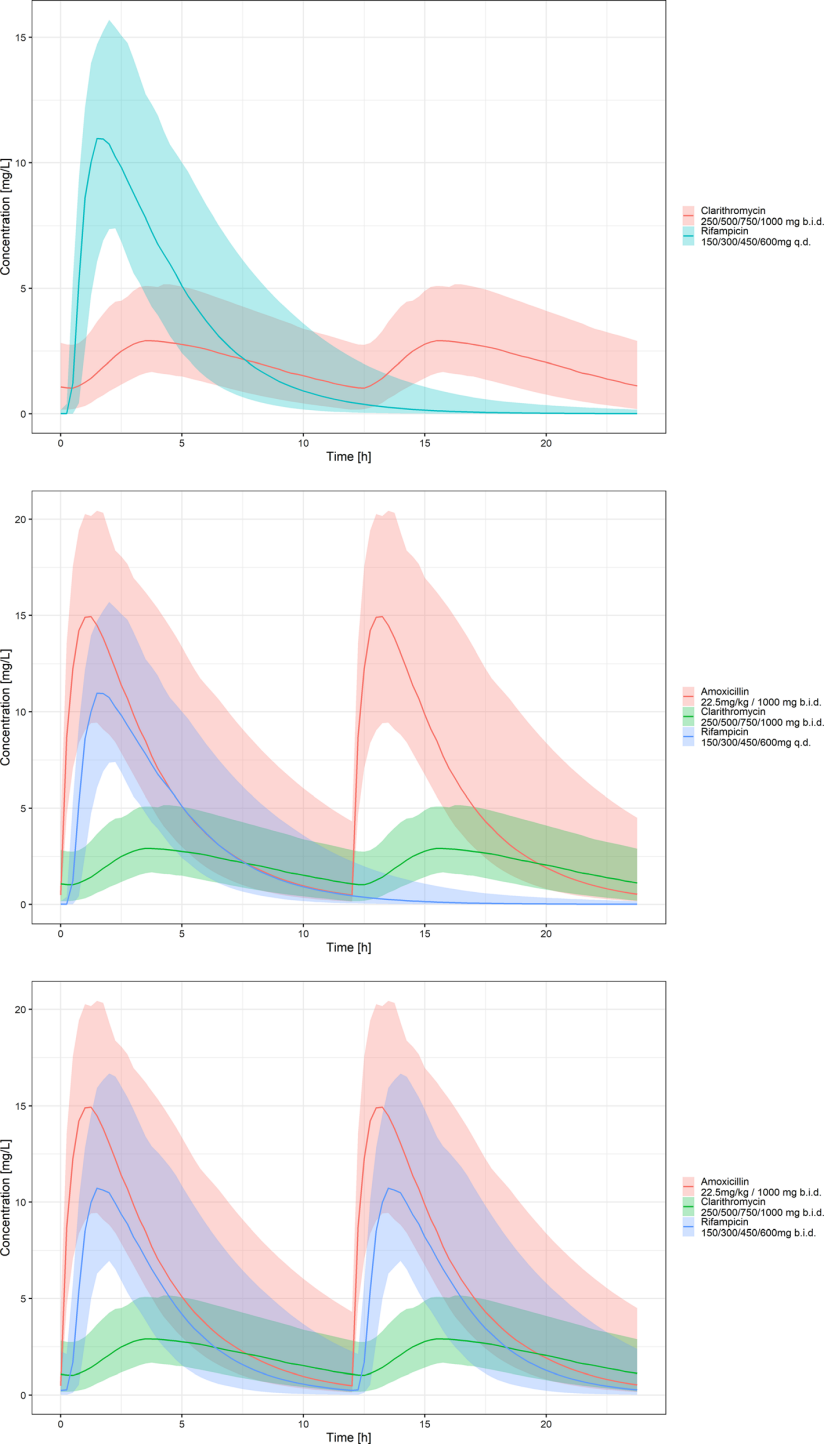
Predicted concentration *vs.* time profiles at steady state following the administration of RIF 150/300/450/600 mg q.d. + CLA 250/500/750/1000 mg b.i.d. + AMX/CLV 22.5 mg/kg or 1000 mg b.i.d. (scenario 1); RIF 75/150/225/300 mg b.i.d. + CLA 250/500/750/1000 mg b.i.d. + AMX/CLV 22.5 mg/kg or 1000 mg b.i.d. (scenario 4) and standard of care regimen *(*scenario 0*)* using weight‐banded doses as described in Table 2 *.* Lines represent the median and shaded areas the 90% prediction intervals. Simulations show results from 1 replicate (*n* = 140), assuming enrolment of adult and paediatric patients according to incidence rates.

Median, 5^th^ and 95^th^ percentiles of the simulated concentration *vs*. time profiles corresponding to each of the drugs used in scenarios 1 and 4 based on strain NCTC 10417 (ATCC19423) are shown in Figure 2. Scenario 1 describes the regimen proposed for study NCT05169554, whereas scenario 4 refers to the regimen yielding the highest PTA. Also shown in Figure 2 is the concentration *vs*. time profile for the standard of care regimen (only including RIF and CLA). As RIF and CLA were administered at the same dose amounts in scenario 3 as scenarios involving RIF and CLA administered at the standard of care doses (scenario 1), these curves provide insight into the overall variability in exposure to the currently used regimens for the treatment of BU. An overview of the PK profiles obtained for the other scenarios is presented in Figures S1 and S2.

The secondary PK parameters derived for the population to be included in the proposed clinical study are summarized (medians, 5^th^ and 95^th^ percentiles) in Table 4. The PK results  for the other scenarios are shown in Table S3.

**TABLE 4:** Predicted total exposure (median, 5^th^ and 95^th^ percentiles) to rifampicin (RIF), clarithromycin (CLA) and amoxicillin/clavulanic acid (AMX/CLV) in patients with BU (n= 67, aged 5‐15 years; n=73 aged >15 years) receiving standard of care doses of RIF and CLA in combination with 22.5 mg/kg or 1000 mg twice daily (b.i.d.) AMX/CLV (scenario 1).  Cavg_ss_ was calculated as AUC_0‐24_/24 h.

Parameter	Standard of care		Standard of care + 22.5 mg/kg or 1000 mg b.i.d. AMX/CLV
	(RIF)	(CLA)	(AMX)
AUC_0‐24_ (mg/L*h)	52.1 (30.7–95.3)	4.4 (2.1–8.9)	108.3 (71.5–224.1)
Cmax_ss_ (mg/L)	11.6 (7.5–15.8)	2.9 (1.7–5.2)	15.2 (9.5–20.8)
Cavg_ss_ (mg/L)	2.2 (1.3–3.9)	0.18 (0.09–0.37)	4.5 (2.9–9.3)

Abbreviations: AUC_0‐24_, area under the concentration vs. time curve over a 24 hour dosing period at steady state; Cavg_ss_, average steady‐state concentration; Cmax_ss_, maximum concentration at steady state.

**FIGURE 3:**
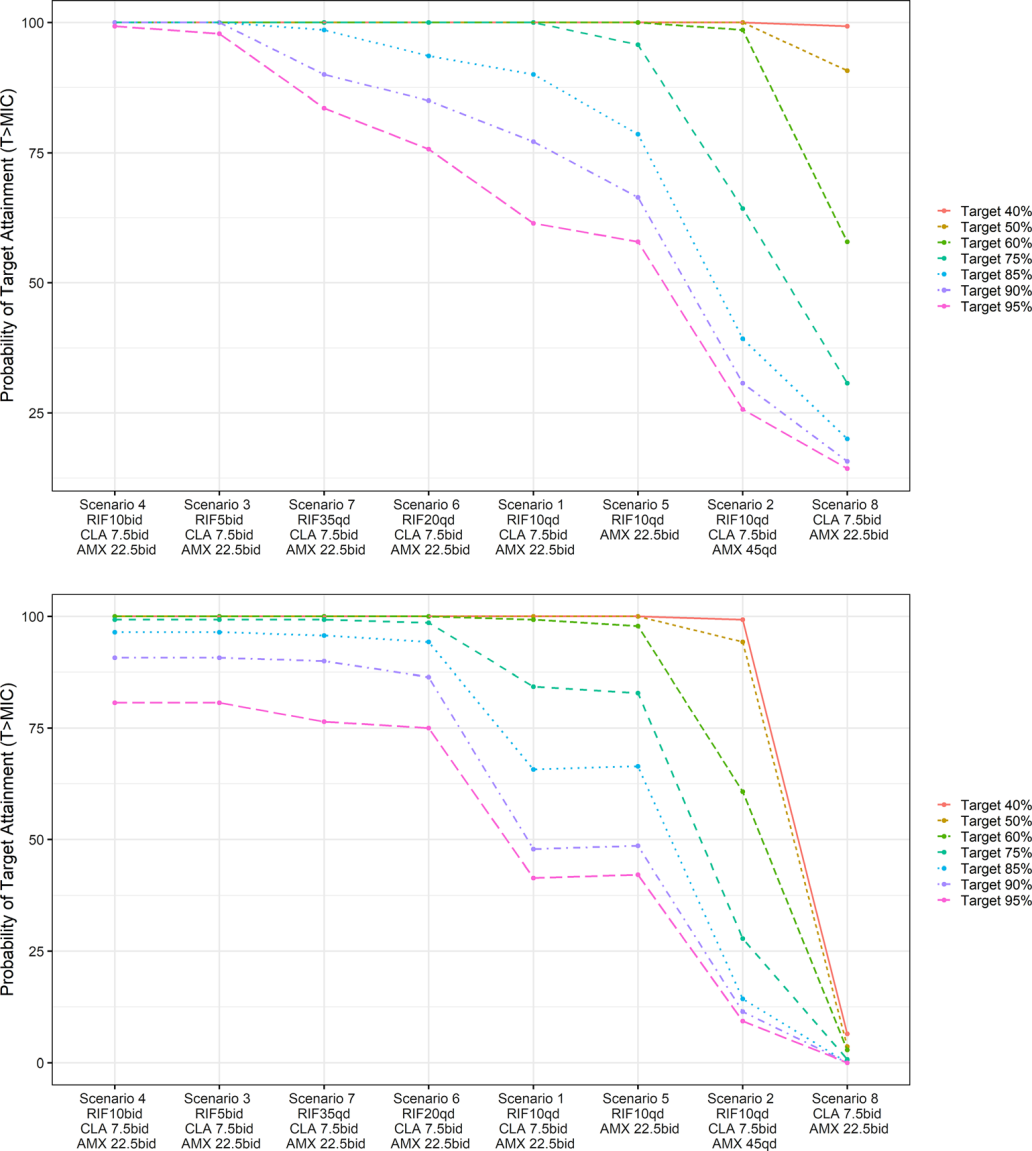
Probability of target attainment (PTA) after administration of amoxicillin/clavulanic acid (AMX/CLV) in combination with rifampicin (RIF) and clarithromycin (CLA). Results are based on systemic unbound fraction (top) and skin (bottom) concentrations of AMX/CLV, susceptibility data from *Mycobacterium ulcerans* strain NCTC 10417 (ATCC19423) and a virtual population of patients affected by BU (n= 67, aged from 5 to 15 years old; n= 73 aged >15 years old), accounting for west African growth parameters and disease incidence (cohort 1). Given the nature of BU infection, PTA has been calculated for different thresholds for the time above the minimum inhibitory concentration (T>MIC), including values >40%. Dosing regimens associated with each scenario are described in Table 2. Median PTA estimates along with 90% prediction intervals from 100 replicates are summarized in Table S6.

The predicted PTA based on *M*. *ulcerans* strain NCTC 10417 (ATCC19423) is summarized graphically for each antibiotic regimen scenario in Figure 3; scenarios 3 and 4 achieved the highest PTA values (PTA > 90%). An overview of the predicted PTA relative to T > MIC of 40% for *M*. *ulcerans* strain NCTC 10417 and each clinical isolate is presented in Table S4. The PTA estimates for the different T > MIC thresholds are listed in Table S5.

Scenario 5, the only scenario excluding CLA from the combination, had a slightly reduced PTA compared to scenarios containing CLA. However, scenario 5 had a significantly higher PTA compared to scenario 8, the only scenario excluding RIF. This finding suggests that the addition of CLA to the triple combination [RIF + AMX/CLV] may have a limited contribution to the overall antibacterial activity, as shown by comparable PTA values, and that the presence of RIF is needed to ensure higher bacterial susceptibility to AMX/CLV, as previously suggested by the in vitro synergy studies.^6^

Clinical isolates ITM 063846, ITM C08756 and ITM C05143, yielded significantly lower PTA values for all antibiotic regimen scenarios, except for scenario 5. ITM C05143 and ITM C08756 also depicted a significantly higher PTA for scenario 8. The different PTA curves obtained for each clinical isolate are depicted in Figure S3.

**FIGURE 4:**
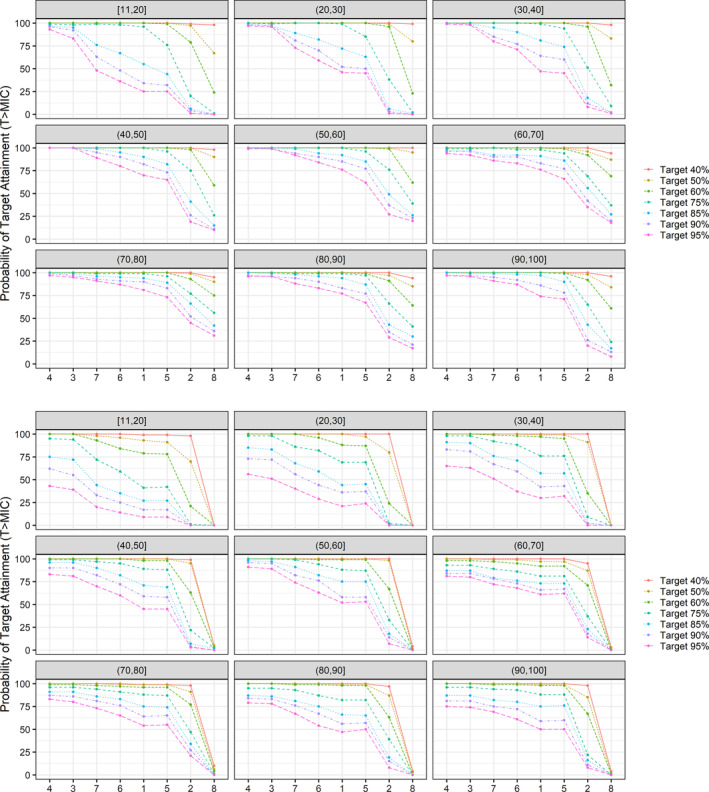
Probability of target attainment after administration of amoxicillin/clavulanic acid in combination with rifampicin and clarithromycin in a virtual population of patients stratified by weight bands (cohort 2). Results are based on *Mycobacterium ulcerans* strain NCTC 10417 (ATCC19423). Probability of target attainment estimates are based on predicted systemic (upper panel) and skin (lower panel) concentrations. Numbers listed on the X‐axis correspond to regimen scenario number. T>MIC, time above the minimum inhibitory concentration. Headers in each panel represent the weight bands (kg). Square brackets and parentheses indicate, respectively, whether the value at the end of the range is included or not within the interval.

Skin tissue exposure to AMX was also considered in our analysis (Figure 3). In this simulation, the PTA decreases for T > MIC threshold above 60%. In addition, given the effect of body weight on drug disposition in paediatric patients, the effect of interindividual variability in exposure on the PTA was evaluated using a larger cohort of patients stratified by weight bands (Figure 4). An overview of the predicted PTA obtained from 100 simulations, along with 90% prediction intervals is shown in Table S6.

These steps allowed for further assessment of the dose rationale for paediatric patients, who are likely to receive AMX/CLV suspension on a mg/kg basis. Scenarios in which AMX/CLV was administered once daily (scenario 2) were shown to have lower PTA for all weight bands in comparison to scenarios in which AMX/CLV was dosed twice daily.

## 4 DISCUSSION

Currently, the recommended treatment of BU relies on empirical evidence of efficacy and safety of a combination of antibiotics and wound care or surgery to support the healing of the ulcer. Whilst the most widely used antibiotic regimen is oral RIF once daily plus CLA twice daily, other combinations have been used sporadically with RIF.^20^ Unfortunately, the empirical choices regarding these alternative combinations have not yielded additional therapeutic advancement.^30^

Recently, in vitro studies^6^ suggested the potential of shortening BU therapy by adding AMX/ CLV to the WHO recommended standard of care treatment and a pragmatic clinical trial is currently under investigation [ClinicalTrials.gov Identifier: NCT05169554; Pan African Clinical Trials Registry Identifier: PACTR202209521256638]. AMX/CLV is normally given three times a day. However, for pragmatic reasons in the resource limited settings in which the trial is being conducted (rural areas in West Africa) and to facilitate patient's adherence, it is being administered twice daily in order to match the posology of CLA. Thus, the question remained as to whether this posology would suffice to ensure appropriate AMX/CLV exposure and if the wider dosing interval could be compensated by the strong in vitro synergistic interaction with RIF and CLA.

Our investigation provides the basis for a more robust rationale for the selection of doses and combinations to be prioritized in a prospective clinical study. Even though the use of quantitative clinical pharmacology principles to translate microbiological findings and integrate in vitro efficacy data with in vivo PK data is not new, such an approach has not been systematically applied to neglected diseases, such as BU.^31^, ^32^, ^33^, ^34^ In fact, these results show that current guidelines do not fully consider the implications of interindividual variability in drug disposition due to body weight.^35^ Dosing in mg/kg assumes that a linear correlation exists between weight and exposure, whereas for most drugs such a correlation is nonlinear.^36^ Consequently, it is conceivable that similar levels of efficacy may not be reached following a shorter treatment, as some patients are exposed to lower drug levels than others. The predicted difference in PTA between paediatric and adult patients with BU was large, particularly in patients with body weight lower than 50 kg.

Based on the different scenarios, it appears that twice daily administration of AMX/CLV is enough and required to achieve the target PTA. This is not possible with once daily administration of AMX/CLV. In addition, the introduction of AMX/CLV and change to the dosing regimen of RIF based on weight bands seems to increase the PTA, overcoming the lower exposure in patients with low body weight. Given that in vitro studies suggest an association between the addition of AMX/CLV and shorter treatment outcome,^6^ addressing the issue of suboptimal exposure is a *sine qua non* condition to ensure the efficacy of shorter treatments.

The potential advantages of a twice daily RIF dosing regimen, possibly with higher doses of RIF were also evaluated. Whilst there may be a preference for once daily regimens in resource‐limited settings, a trade‐off between enhanced antibacterial activity and treatment duration needs to be considered. As shown in Figure S2, an increase in the dosing frequency will provide adequate conditions for the synergistic interaction between the components within the combination. The proposed increase in the doses of RIF is not expected to lead to a different safety profile, as recently observed in clinical trials in tuberculosis, with patients receiving higher doses of RIF (up to 35 mg/kg body weight).^37^, ^38^ Moreover, it is plausible to assume that a novel regimen (RIF twice daily) will not affect the overall adherence to treatment, given that CLA is already used twice daily and AMX/CLV also needs to be administered twice daily to ensure appropriate exposure levels.

Another interesting finding was that the predicted PTA was usually higher for antibiotic combinations excluding CLA (scenario 5) than combinations excluding RIF. Whilst these differences may not be statistically significant, this presents an opportunity to explore simplified regimens with RIF and AMX/CLV only. Analysis of regimen scenarios using a combination of AMX/CLV with CLA in absence of RIF (scenario 8) showed the lowest PTA of all assessed regimens, reinforcing the importance of the contribution of RIF in treatment combination with AMX/CLV. These results are of special importance in the case of patients who cannot tolerate CLA. One of the potential side effects of CLA therapy is prolongation of the QT interval.^39^, ^40^ This can be life threatening in patients with pre‐existing cardiopathies. In these cases, switching to the previously recommended therapy is indicated, i.e., replacing CLA by streptomycin (STR) injections. However, long‐term use of STR induces ototoxicity and injections are painful. In addition, in most rural settings STR is not readily available since it is no longer provided by the WHO to the BU National Control programmes. Thus, the combination of RIF with AMX/CLV gives an alternative treatment option to these patients, without increasing the risk of potential development of resistance with RIF monotherapy.

There are several areas in which our study presents some limitations, regarding the PK modelling of the different antibiotics and translation of the effects observed in vitro. Similarly, we have not been able to explore all relevant baseline characteristics of the virtual population used for simulations. Further details on these limitations can be found in the Supporting Information.

In conclusion, the higher PTA values support the proposed combination (RIF, CLA, AMX/CLV) currently under clinical investigation. Our findings also suggest that the use of CLA is dispensable and that higher RIF doses, together with AMX/CLV, might contribute to enhanced efficacy. However, in order to further evaluate the impact of the observed pharmacodynamic interaction, efforts to establish drug distribution into the skin should be considered in conjunction with physiologically based PK modelling.

## AUTHOR CONTRIBUTION

S.D.A., P.V. and N.G.B. performed the analysis and contributed to manuscript writing, S.D.A., S.R.G. and O.D.P. contributed to the conceptualization, research objectives, interpretation of results and manuscript writing.

## CONFLICTS OF INTEREST STATEMENT

All authors declare no conflict of interest. O.D.P. is also an employee of GSK R&D.

## Supporting information


**TABLE S1** Details of the growth parameters in West African children, taken from a cross sectional study of children, aged 6–18 years in Calabar, South Nigeria. A total of 2830 subjects were recruited for the study.
**TABLE S2** Doses (mg or mg/kg) and dosing regimen (q.d. or b.i.d.) proposed for rifampicin (RIF), clarithromycin (CLA) and amoxicillin/clavulanic acid (AMX/CLV) in the clinical studies. Adults and paediatric patients weighing >40 kg body weight receive a solid dosage form (tablets), whilst those with *≤*40 kg receive AMX/CLV as a suspension.
**TABLE S3** Secondary PK parameters of RIF and AMX/CLV following dosing regimens used across the simulated scenarios, which were not included in Table 4 (main manuscript). Values are median, 5^th^ and 95^th^ percentiles. Cavg_ss_ was calculated as AUC_0‐24_ /24 hours.
**TABLE S4** Effect of varying bacterial susceptibility on the PTA for T > MIC of 40% using the prospective protocol scenario in a trial setting with Cohort 1. Details of the doses and dosing regimens used in each scenario are shown in Table 2 in the manuscript.
**TABLE S5** Effect of different thresholds for T > MIC, namely 40, 50, 60, 75, 65, 90 and 95% on the PTA. Details of the doses and dosing regimens used in each scenario are shown in Table 2 in the manuscript. Results reflect PTA values for *M. ulcerans* strain NCTC 10417 (ATCC19423) in a trial setting with Cohort 1.
**TABLE S6** Median and 90% prediction intervals of the PTA for different thresholds for T > MIC, namely 40, 50, 60, 75, 65, 90 and 95%. Details of the doses and dosing regimens used in each scenario are shown in Table 2 in the manuscript. Results reflect PTA values for *M. ulcerans* strain NCTC 10417 (ATCC19423) in a trial setting with Cohort 1 based on 100 replicates.
**FIGURE S1** Predicted concentration *vs.* time profile at steady state following the administration of scenario 2 (RIF 150/300/450/600 mg q.d. + CLA 250/500/750/1000 mg b.i.d. + AMX/CLV 45 mg/kg or 2000 mg q.d.), scenario 5 (RIF 150/300/450/600 mg q.d. + AMX/CLV 22.5 mg/kg or 1000 mg b.i.d.), scenario 6 (RIF 300/600/900/1200 mg q.d. + CLA 250/500/750/1000 mg b.i.d. + AMX/CLV 22.5 mg/kg or 1000 mg b.i.d.), scenario 7 (RIF 525/1050/1575/2100 mg q.d. + CLA 250/500/750/1000 mg b.i.d. + AMX/CLV 22.5 mg/kg or 1000 mg b.i.d.), scenario 8 (CLA 250/500/750/1000 mg b.i.d. + AMX/CLV 22.5 mg/kg or 1000 mg b.i.d.).
**FIGURE S2** Concentration vs. time profiles at steady state for scenarios including rifampicin as a twice daily regimen. Upper panel, scenario 3 (RIF 75/150/225/300 mg b.i.d. + CLA 250/500/750/1000 mg b.i.d. + AMX/CLV 22.5 mg/kg or 1000 mg b.i.d). Lower panel, scenario 4 (RIF 150/300/450/600 mg b.i.d. + CLA 250/500/750/1000 mg b.i.d. + AMX/CLV 22.5 mg/kg or 1000 mg b.i.d.). Lines represent the median and shaded areas are 95% prediction intervals.
**FIGURE S3** Effect of varying bacterial susceptibility on the probability of target atainment after administration of AMX/CLV in combination with RIF and CLA. Each panel depicts a different clinical isolate of M. ulcerans. Results correspond to simulations including a virtual population (n= 67, aged from 5 to 15 years old; n= 73 aged >15 years old) of patients with BU, taking into account the West African growth curves and disease prevalence. Scenarios are presented in descending order of probability of target atainment (PTA).

## Data Availability

The data that support the findings of this study are available from the corresponding author upon reasonable request.
