# Supplementary material for: Optimizing β‐lactam‐containing antibiotic combination therapy for the treatment of Buruli ulcer
Source: Br J Clin Pharmacol. 2024 Sep 18;91(1):179–89. doi: 10.1111/bcp.16209 (PMC11671329; doi:10.1111/bcp.16209)
Supplement: Supplementary file 1 — TABLE S1 Details of the growth parameters in West African children, taken from a cross sectional study of children, aged 6–18 years in Calabar, South Nigeria. A total of 2830 subjects were recruited for the study. TABLE S2 Doses (mg or mg/kg) and dosing regimen (q.d. or b.i.d.) proposed for rifampicin (RIF), clarithromycin (CLA) and amoxicillin/clavulanic acid (AMX/CLV) in the clinical studies. Adults and paediatric patients weighing >40 kg body weight receive a solid dosage form (tablets), whilst those with ≤40 kg receive AMX/CLV as a suspension. TABLE S3 Secondary PK parameters of RIF and AMX/CLV following dosing regimens used across the simulated scenarios, which were not included in Table 4 (main manuscript). Values are median, 5th and 95th percentiles. Cavgss was calculated as AUC0‐24 /24 hours. TABLE S4 Effect of varying bacterial susceptibility on the PTA for T > MIC of 40% using the prospective protocol scenario in a trial setting with Cohort 1. Details of the doses and dosing regimens used in each scenario are shown in Table 2 in the manuscript. TABLE S5 Effect of different thresholds for T > MIC, namely 40, 50, 60, 75, 65, 90 and 95% on the PTA. Details of the doses and dosing regimens used in each scenario are shown in Table 2 in the manuscript. Results reflect PTA values for M. ulcerans strain NCTC 10417 (ATCC19423) in a trial setting with Cohort 1. TABLE S6 Median and 90% prediction intervals of the PTA for different thresholds for T > MIC, namely 40, 50, 60, 75, 65, 90 and 95%. Details of the doses and dosing regimens used in each scenario are shown in Table 2 in the manuscript. Results reflect PTA values for M. ulcerans strain NCTC 10417 (ATCC19423) in a trial setting with Cohort 1 based on 100 replicates. FIGURE S1 Predicted concentration vs. time profile at steady state following the administration of scenario 2 (RIF 150/300/450/600 mg q.d. + CLA 250/500/750/1000 mg b.i.d. + AMX/CLV 45 mg/kg or 2000 mg q.d.), scenario 5 (RIF 150/300/450/600 mg q.d. [file BCP-91-179-s001.pdf]

Supporting Information

Table S1. Details of the growth parameters in west African children, taken from a cross sectional study of children, aged 6-18 years in Calabar, South Nigeria. A total of 2830 subjects were recruited for the study.

**Boys**				**Girls**			
**Age (year)**	**Mean Weight (kg)**	**Mean Heigh (cm)**	**BMI (kg/m^2)**	**Age (year)**	**Mean Weight (kg)**	**Mean Height (cm)**	**BMI (kg/m^2)**
**6**	21.9	117.6	15.7	**6**	20.9	116.0	15.5
**7**	26.8	126.8	16.6	**7**	26.1	128.2	15.8
**8**	28.9	131.9	16.6	**8**	27.6	129.8	16.2
**9**	31.0	136.5	16.6	**9**	34.9	138.1	18.0
**10**	34.2	140.1	17.5	**10**	34.8	143.1	16.9
**11**	36.0	144.5	17.1	**11**	39.4	146.8	18.0
**12**	38.9	148.1	17.6	**12**	43.2	152.5	18.6
**13**	42.6	153.3	17.9	**13**	45.7	154.2	19.0
**14**	45.5	157.8	18.2	**14**	50.9	156.8	20.6
**15**	50.7	160.8	19.7	**15**	52.0	159.2	20.2
**16**	57.1	167.6	20.3	**16**	51.6	156.2	21.2
**17**	57.2	168.3	20.1	**17**	51.6	155.1	21.4
**18**	58.2	165.6	21.2	**18**	61.5	161.5	23.5

Table S2. Doses (mg or mg/kg) and dosing regimen (q.d. or b.i.d.) proposed for rifampicin (RIF), clarithromycin (CLA) and amoxicillin/clavulanic acid (AMX/CLV) in the clinical studies. Adults and paediatric patients weighing >40 kg body weight receive a solid dosage form (tablets), whilst those with ≤40 kg receive a AMX/CLV as a suspension.

Posology for rifampicin and clarithromycin in studies NCT05169554 and PACTR202209521256638

Patient's body weight (kg)	Rifampicin [150 or 300 mg / capsule] once daily			Clarithromycin [250 mg or 500 mg / tablet] twice daily				
	Dose (mg)	No. of capsules [150 mg]	No. of capsules [300 mg]	Daily dose (mg)	No. of tablets [250 mg] per dose	No. of tablets [250 mg] (total daily)	No. of tablets [500 mg] per dose	No. of tablets [500 mg] (total daily)
**11-≤20**	150	1	NA	250	0.5	1	-	-
**>20-≤40**	300	-	1	500	1	2	-	-
**>40-≤54**	450	1	1	750	1	1	1	1
**>54**	600	-	2	1000	-	-	1	2

Posology for amoxicillin/clavulanic acid in studies NCT05169554 and PACTR202209521256638

[1]Patient's body weight (kg)	[2]Dose (mg)	[2]Total daily dose (mg)	[3]Syrup [100/12.5 mg/mL] (mL/dose)	[3]Syrup [100/12.5 mg/mL] (mL) (total daily)	[4]No. of tablets [500/62.5 mg] per dose	[4]No. of tablets [500/62.5 mg] (total daily)
**11 - 12**	270	540	2.7	5.4	NA	NA
**13 - 14**	315	630	3.1	6.2	NA	NA
**15 - 16**	360	720	3.6	7.2	NA	NA
**17 - 18**	405	810	4	8	NA	NA
**19 - 20**	450	900	4.5	9	NA	NA
**21 - 22**	495	990	4.9	9.8	NA	NA
**23 - 24**	540	1080	5.4	10.8	NA	NA
**25 - 26**	585	1170	5.8	11.6	NA	NA
**27 - 28**	630	1260	6.3	12.6	NA	NA
**29 - 30**	675	1350	6.8	13.6	NA	NA
**31 - 32**	720	1440	7.2	14.4	NA	NA
**33 - 34**	765	1530	7.7	15.4	NA	NA
**35 - 36**	810	1620	8.1	16.2	NA	NA
**37 - 38**	855	1710	8.5	17	NA	NA
**39 - 40**	900	1800	9	18	NA	NA
**>40**	1000	2000	NA	NA	2	4

[1] Weights were arranged in groups of 2 kg. Dose will be adjusted according to the closest body weight group, e.g., if the patient weighs 12.4 kg, dose will be the one corresponding to 12 kg; if the patient weighs 12.5 kg or above, the dose will be that one for 13 kg.

[2]Dose of AMX/CLV 1000/125 mg twice daily, which makes a total of 2000/250 mg/day, for patients over 40 kg, and 22.5/5.6 mg/kg twice daily, which makes a total of 45/11.25 mg/kg/day, for those equal and below 40 kg. For children, posology will be adapted to the age of the patient according to drug manufacturer indications. There are no current recommendations for the use of AMX/CLV for BU infections. Those indicated above are recommended doses for complicated infections according to Augmentin's manufacturer indications.

[3] Formulations of AMX/CLV syrup are available (250/31.25 mg for 5 ml). For a bottle of 75 mL, 100 mL or 150 mL, the necessary quantity of water for reconstitution would be 65 mL, 87 mL and 130 mL, respectively. The syrup will be reconstituted just before the dose administration. Doses will be administered with graduated syringes.

[4] Tablets of AMX/CLV are available (500/62.5 mg)

Table S3. Secondary PK parameters of RIF and AMX/CLV following dosing regimens used across the simulated scenarios, which were not included in Table 4 (main manuscript). Values are median, 5th and 95th percentiles. $Cavg_{ss}$ was calculated as AUC_{0-24}/24 h.

Parameter	RIF 5mg/kg b.i.d.	RIF 10mg/kg b.i.d.	RIF 20mg/kg q.d.	RIF 35 mg/kg q.d.	AMX 45mg/kg q.d.
AUC_{0-24} (mg/L*h)	42.4 (24.2-86.2)	89.8 (49.8-186.9)	116.8 (68.2- 225.4)	239.7 (136.1-502.9)	108.4 (71.5-224.1)
$Cmax_{ss}$ (mg/L)	5.4 (3.6-8.1)	11.0 (7.3-16.7)	23.5 (15.8-32.7)	42.7 (28.9-60.1)	29.2 (18.3-38.6)
$Cavg_{ss}$ (mg/L)	1.7 (1.0-3.6)	3.7 (2.1-7.8)	4.8 (2.8-9.4)	9.9 (5.7-20.9)	4.5 (2.9-9.3)

Table S4. Effect of varying bacterial susceptibility on the PTA for T> MIC of 40% using the prospective protocol scenario in a trial setting with Cohort 1. Details of the doses and dosing regimens used in each scenario are shown in Table 2, in the manuscript.

Isolate	Scenario 1	Scenario 2	Scenario 3	Scenario 4	Scenario 5	Scenario 6	Scenario 7	Scenario 8
**ATCC19423**	100	100	100	100	100	100	100	99.2
**ITM 063846**	100	89.28	100	100	100	100	100	70
**ITM C05142**	100	100	100	100	100	100	100	100
**ITM C05143**	75	25.7	70.7	70	98.6	74.2	70.7	98.6
**ITM C08756**	100	100	100	100	100	100	100	100
**ITM M000932**	100	99.2	100	100	100	100	100	99.2

Table S5. Effect of different thresholds for T> MIC, namely 40, 50, 60, 75, 65, 90 and 95% on the PTA. Details of the doses and dosing regimens used in each scenario are shown in **Table 2** in the manuscript. Results reflect PTA values for *M. ulcerans* strain NCTC 10417 (ATCC19423) in a trial setting with Cohort 1.

Threshold for T>MIC	Scenario 1	Scenario 2	Scenario 3	Scenario 4	Scenario 5	Scenario 6	Scenario 7	Scenario 8
**40%**	100	100	100	100	100	100	100	99.2
**50%**	100	100	100	100	100	100	100	90.7
**60%**	100	98.5	100	100	100	100	100	57.8
**75%**	100	64.2	100	100	96.4	100	100	31.4
**85%**	90	39.2	100	100	79.2	93.5	98.5	20
**90%**	77.1	30.7	100	100	67.1	85.7	90	15.7
**95%**	62.1	25.7	98.5	99.2	58.5	76.4	83.5	14.2

Table S6. Median and 90% prediction intervals of the PTA for different thresholds for T> MIC, namely 40, 50, 60, 75, 65, 90 and 95%. Details of the doses and dosing regimens used in each scenario are shown in **Table 2** in the manuscript. Results reflect PTA values for *M. ulcerans* strain NCTC 10417 (ATCC19423) in a trial setting with Cohort 1 based on 100 replicates.

Threshold for T>MIC	Scenario 1	Scenario 2	Scenario 3	Scenario 4	Scenario 5	Scenario 6	Scenario 7	Scenario 8
**40%**	100 (100-100)	100 (100-100)	100 (100-100)	100 (100-100)	100 (100-100)	100 (100-100)	100 (100-100)	97.9 (95.7-99.3)
**50%**	100 (100-100)	99.2 (97.8-100)	100 (100-100)	100 (100-100)	100 (100-100)	100 (100-100)	100 (100-100)	86.4 (81.4-90.7)
**60%**	100 (100-100)	94.6 (90.7-97.85)	100 (100-100)	100 (100-100)	100 (99.3-100)	100 (100-100)	100 (100-100)	55 (47.1-60.7)
**75%**	99.3 (98.5-100)	62.5 (57.1-68.6)	100 (99.3-100)	100 (99.9-100)	95.7 (92.1-98.6)	100 (99.3-100)	100 (99.3-100)	27.1 (22.1-32.9)
**85%**	87.9 (83.6-92.2)	37.1 (30-42.9)	99.3 (97.9-100)	100 (98.6-100)	79.3 (74.2-85)	93.2 (89.9-97.1)	96.4 (93.6-99.3)	17.9 (13.6-22.9)
**90%**	77.1 (71.4-82.1)	28.6 (22.1-32.8)	98. 6 (96.4-100)	99.3 (97.9-100)	70 (64.9-77.2)	85 (80.7-90)	91.4 (87.9-95)	15 (11.4-19.3)
**95%**	65.7 (59.3-71.4)	20.7 (15.7-25)	97.1 (94.3-99.3)	98.6 (96.4-100)	60.7 (54.6-68.6)	76.4 (69.9-82.1)	85 (80.7-90.7)	12.9 (9.3-16.4)

Scenario 2

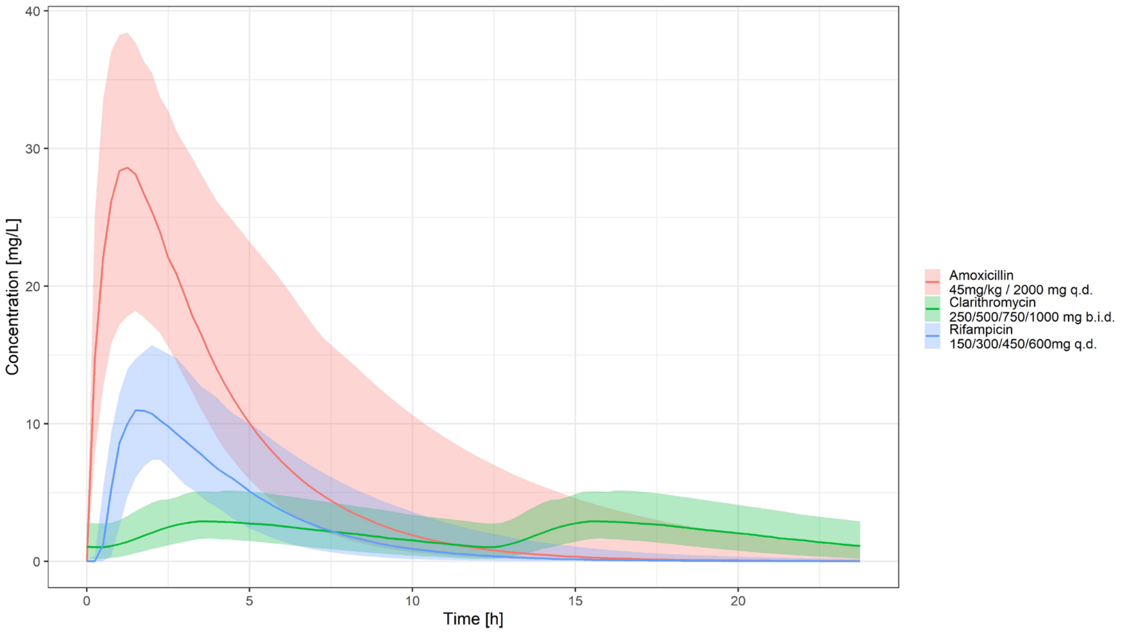

Scenario 5

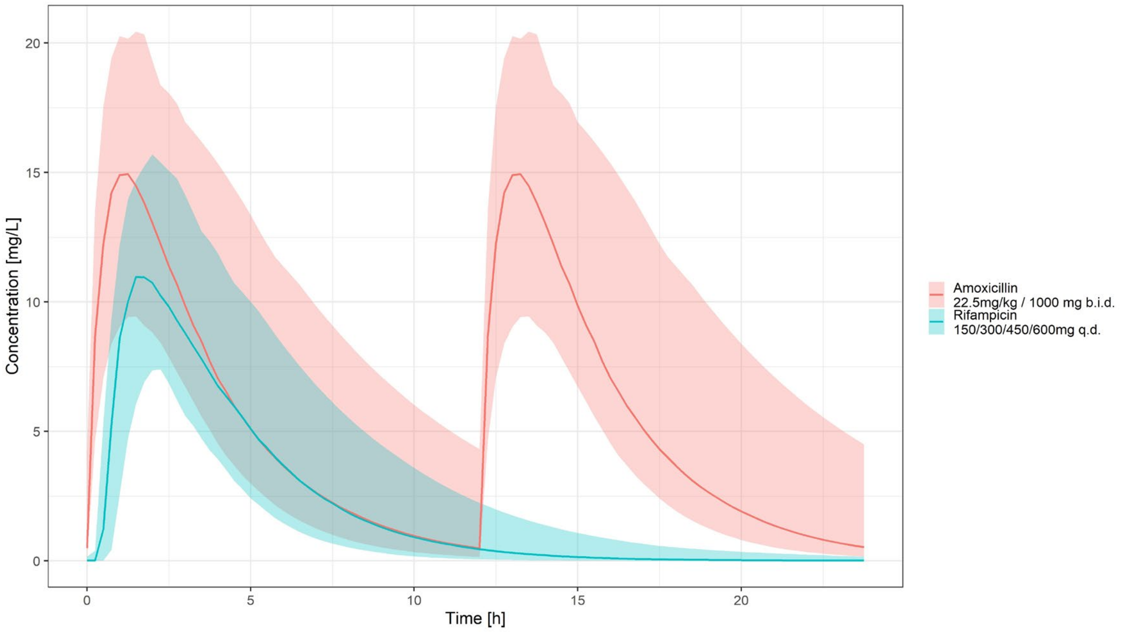

Scenario 6

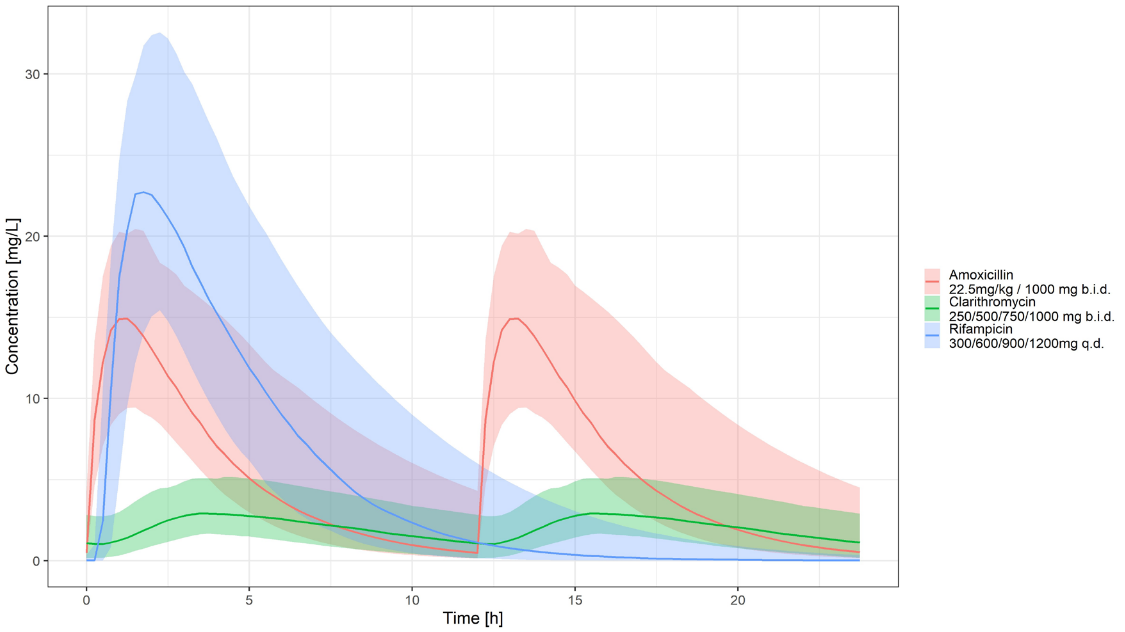

Scenario 7

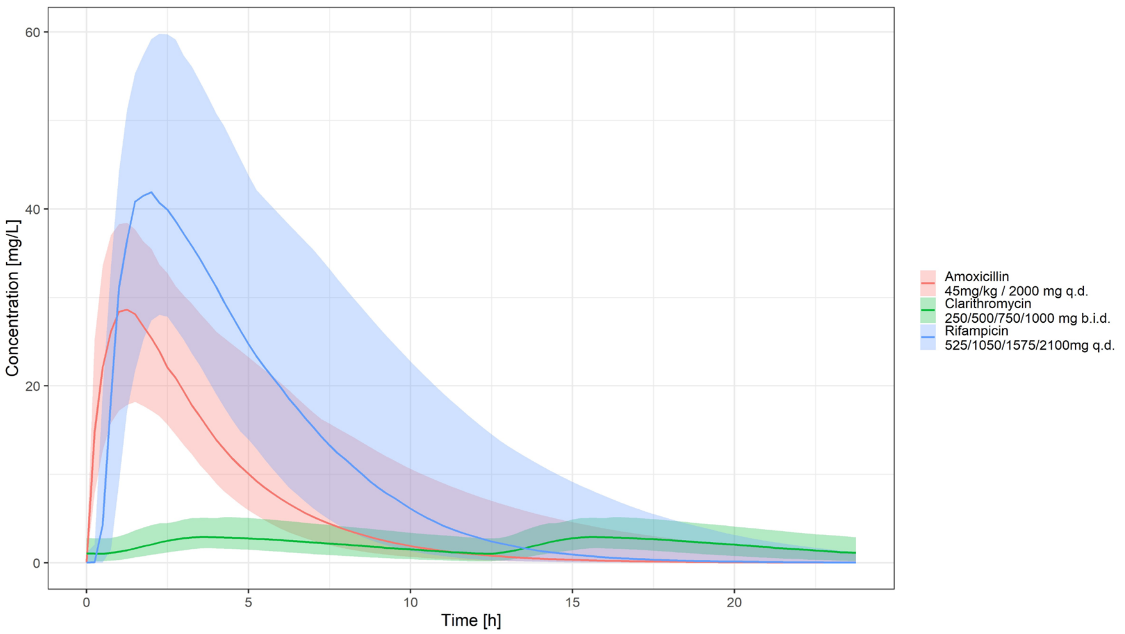

Scenario 8

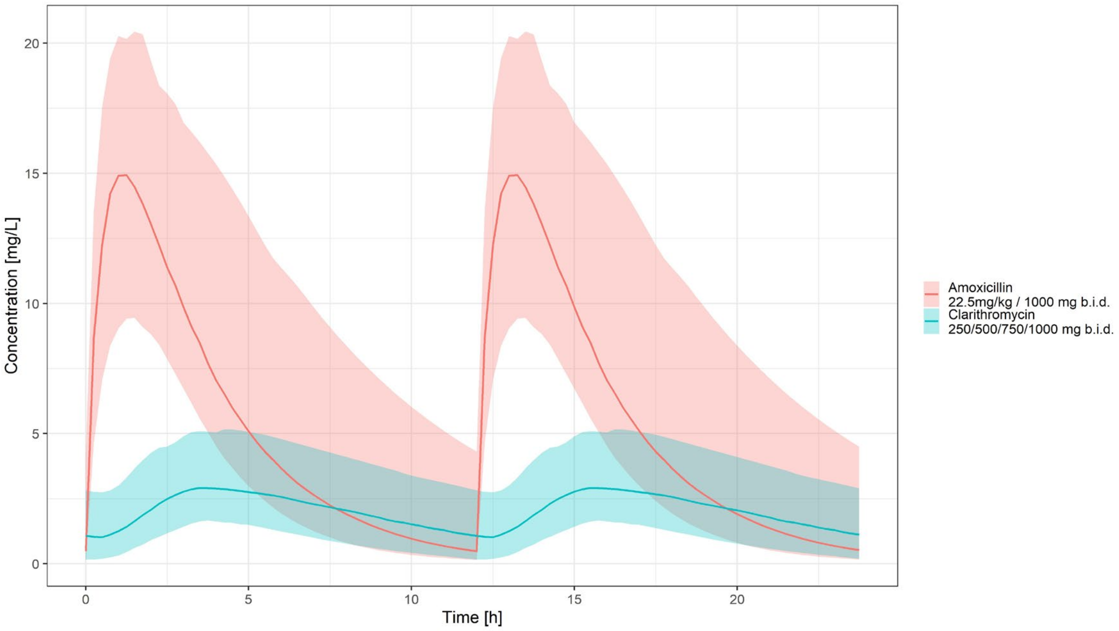

Figure S1. Predicted concentration vs time profiles at steady state following the administration different doses and combinations, as defined in **scenario 2** (RIF 150/300/450/600 mg q.d. + CLA 250/500/750/1000 mg b.i.d. + AMX/CLV 45 mg/kg or 2000 mg q.d.), **scenario 5** (RIF 150/300/450/600 mg q.d. + AMX/CLV 22.5 mg/kg or 1000 mg b.i.d.), **scenario 6** (RIF 300/600/900/1200 mg q.d. + CLA 250/500/750/1000 mg b.i.d. + AMX/CLV 22.5 mg/kg or 1000 mg b.i.d.), **scenario 7** (RIF 525/1050/1575/2100 mg q.d. + CLA 250/500/750/1000 mg b.i.d. + AMX/CLV 22.5 mg/kg or 1000 mg b.i.d.), and **scenario 8** (CLA 250/500/750/1000 mg b.i.d. + AMX/CLV 22.5 mg/kg or 1000 mg b.i.d.).

Scenario 3

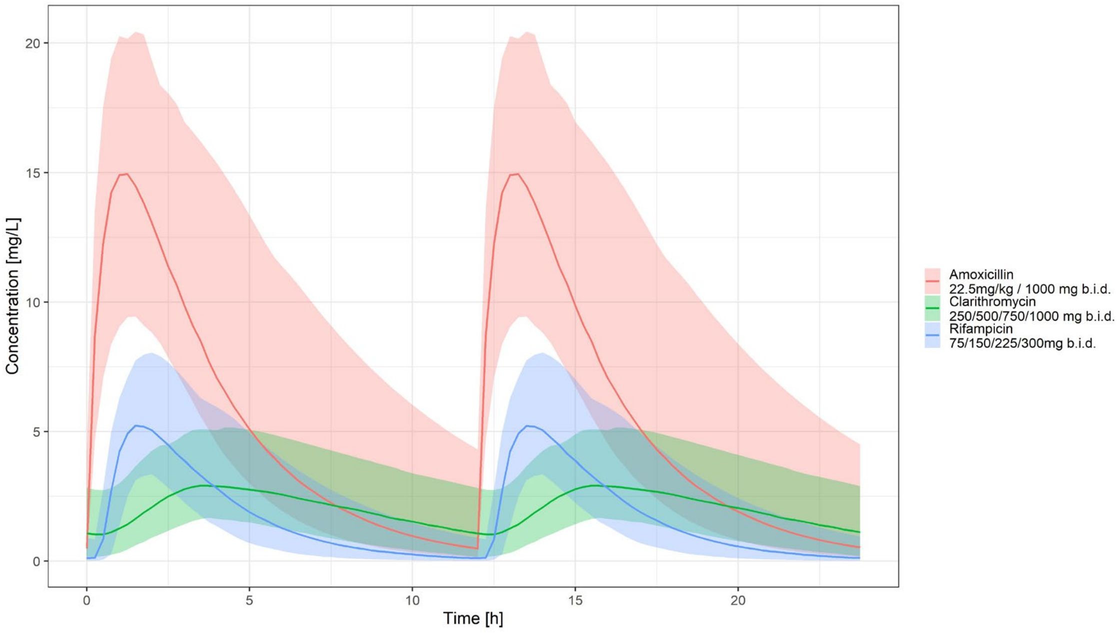

Scenario 4

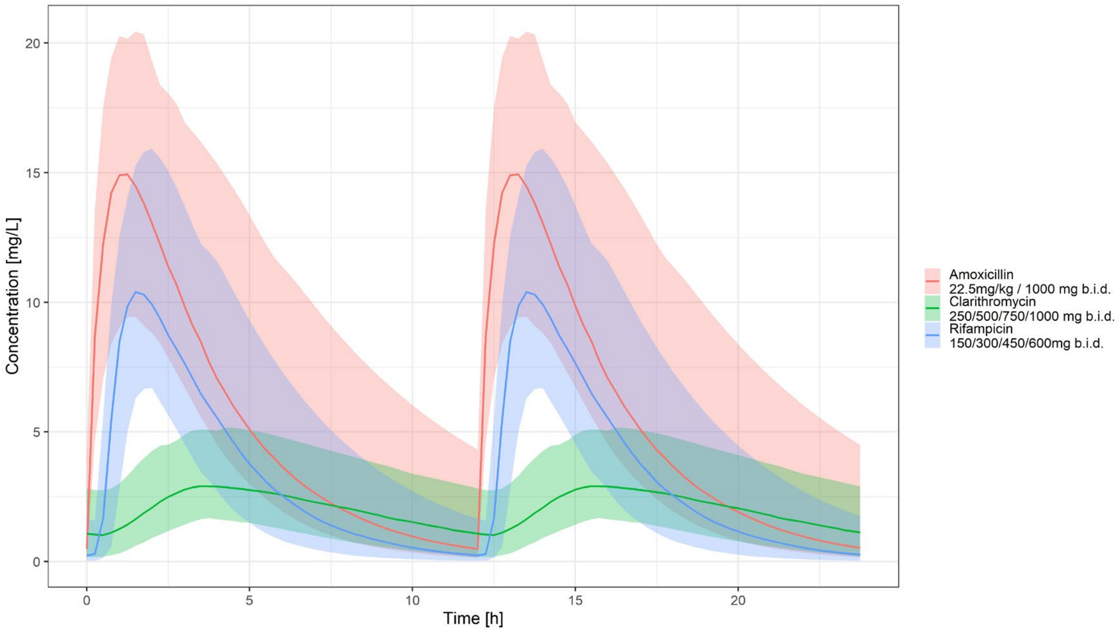

Figure S2. Concentration vs time profiles at steady state for scenarios including rifampicin as a twice daily dosing regimen. Upper panel, **scenario 3 (**RIF 75/150/225/300 mg b.i.d. + CLA 250/500/750/1000 mg b.i.d. + AMX/CLV 22.5 mg/kg or 1000 mg b.i.d). Lower panel, **scenario 4** (RIF 150/300/450/600 mg b.i.d. + CLA 250/500/750/1000 mg b.i.d. + AMX/CLV 22.5 mg/kg or 1000 mg b.i.d.). Lines represent the median and shaded areas are 95% prediction intervals.

ITM063846

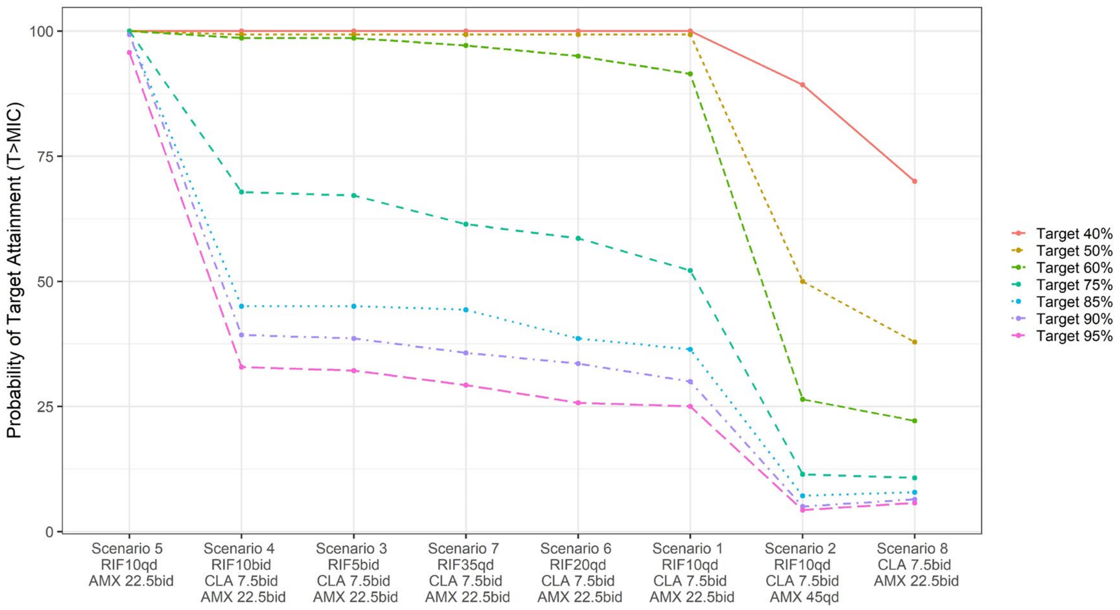

ITMC05142

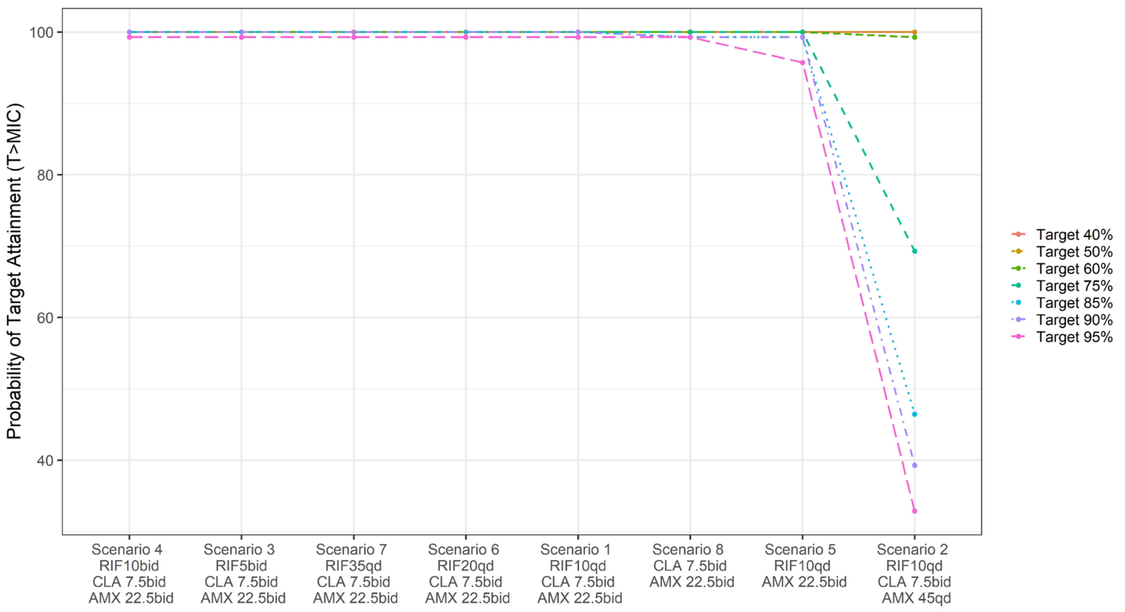

ITMC05143

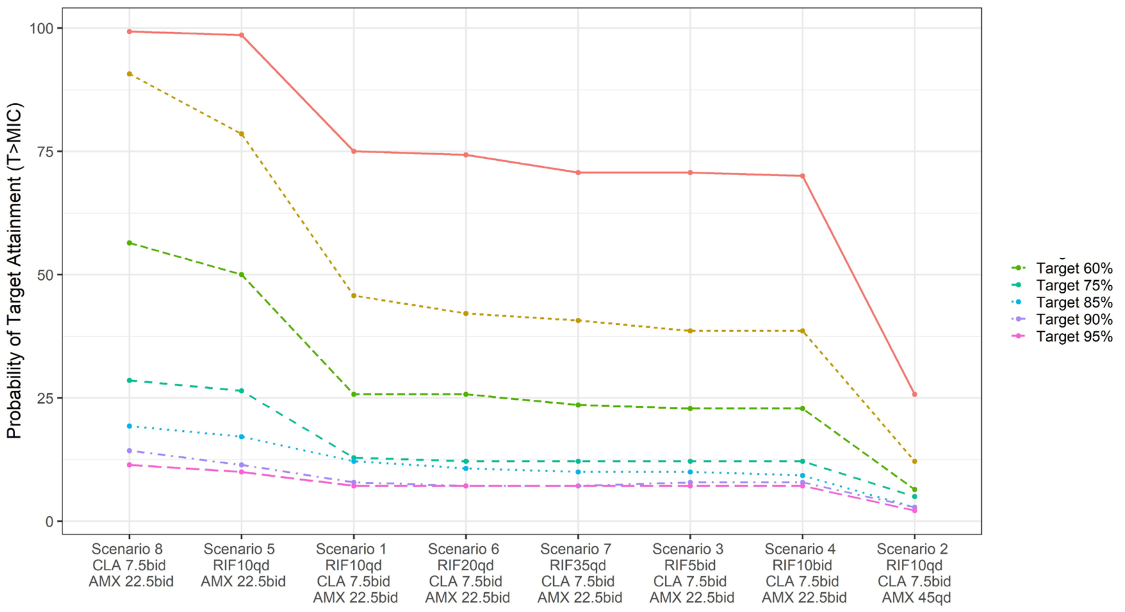

ITMC08756

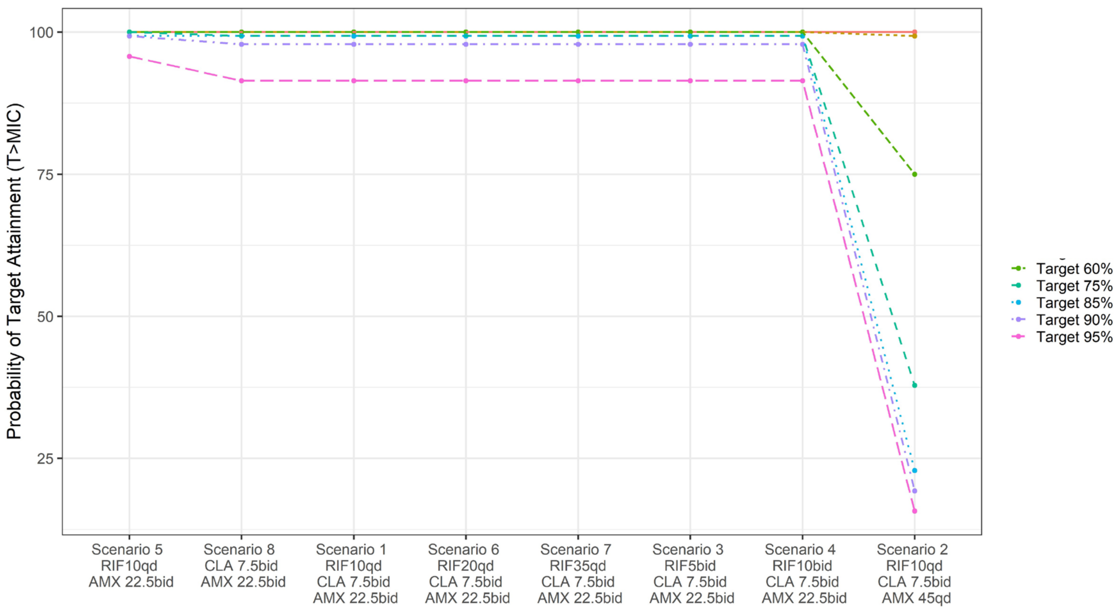
Probability of Target Attainment (T>MIC)
100
75
50
25
Scenario 5
RIF10qd
AMX 22.5bid
Scenario 8
CLA 7.5bid
AMX 22.5bid
Scenario 1
RIF10qd
CLA 7.5bid
AMX 22.5bid
Scenario 6
RIF20qd
CLA 7.5bid
AMX 22.5bid
Scenario 7
RIF35qd
CLA 7.5bid
AMX 22.5bid
Scenario 3
RIF5bid
CLA 7.5bid
AMX 22.5bid
Scenario 4
RIF10bid
CLA 7.5bid
AMX 22.5bid
Scenario 2
RIF10qd
CLA 7.5bid
AMX 45qd
Target 60%
Target 75%
Target 85%
Target 90%
Target 95%

ITMM000932

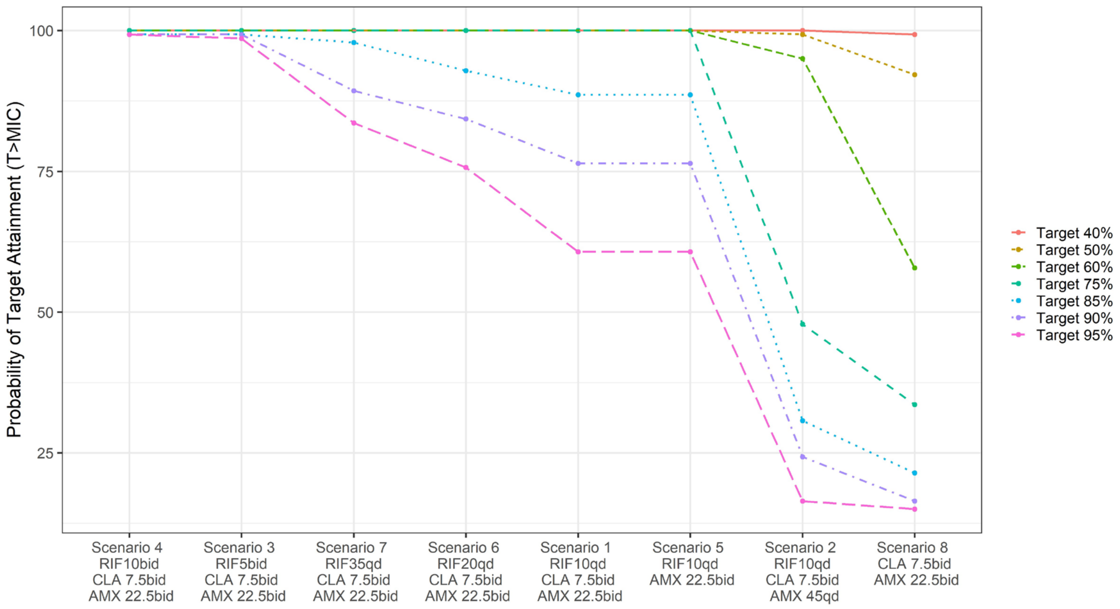

Figure S3. Effect of varying bacterial susceptibility on the probability of target attainment after administration of AMX/CV in combination with RIF and CLA. Each panel depicts a different clinical isolate of *M. ulcerans.* Results correspond to simulations including a virtual population (n= 67, aged from 5 to 15 years old; n= 73 aged >15 years old) of BU patients taking into account the West African growth curves and disease prevalence. Scenarios are presented in descending order of probability of target attainment.

Supplementary Information: Limitations

Our analysis presents several limitations. First, it should be acknowledged that published PK models for AMX were obtained from relatively small studies, with different formulations than the ones which may be required for a prospective study in adult and paediatric patients. Even though there are not many factors known to influence the disposition of AMX, other than renal function, the analysis is based primarily on systemic exposure. Given the nature of the lesions, and necrotic tissue formation, one cannot exclude very slow equilibration kinetics, with variable levels of drug at the site of infection relative to plasma. As no data are available on the pharmacokinetics of AMX/CV in human skin, our analysis has relied on the assumption that the same distribution characteristics observed in rabbits can be applied to humans [1]. Hence, the same plasma-to-tissue ratio was used to establish the PTA in the different simulation scenarios. Another important point is the known instability of CV in acidic medium, and importance of timing of dosing relative to food intake. Whilst the pharmacokinetics of CV was not considered during the simulations, the predicted PTA for AMX assumes that adequate concentrations of CV are maintained throughout the dosing interval [2,3].

From a pharmacodynamic perspective, we have also had to assume that time above the MIC is the best descriptor of the antibacterial activity of the combination, as AMX/CV is the only component associated with a time-dependent mechanism of action. Hence, achievement of higher PTA is directly linked to a longer period exposed to pharmacologically active levels of AMX. In this context, we also acknowledge that optimisation of the doses of RIF and CLA was not within the scope of our investigation. The proposed higher dose of RIF was based on prior clinical judgment based on other indications. In fact, this RIF regimen has been selected for the BuruliRIFDACC study, aimed at the evaluation of high dose rifampicin and dialkylcarbamoyl chloride (DACC)-coated dressings to improve outcomes in *Mycobacterium ulcerans* disease (PACTR202011867644311).

Clearly, the observed variation in PTA across the different clinical isolates indicated an opportunity for further optimisation of the doses. It is also conceivable that the higher PTA values obtained for the different clinical isolates treated in which CLA was not included may be explained by the known drug-drug interaction (i.e., CYP450 induction) between RIF and CLA. Our pharmacokinetic analysis included a term describing the interaction between RIF and CLA, which leads to a decrease in CLA concentrations of up to 67% and increased or variable concentrations of 14-hydroxy-CAM (M-5) metabolite [4,5].

Lastly, although the extent of distribution between plasma and skin of amoxicillin (AMX) was considered, similar analysis was not conducted for rifampicin or clarithromycin. Based on the

reported partition coefficient for skin interstitial fluid and plasma in rodents (0.76 for rifampicin and 1.2 for clarithromycin) [6, 7], there may be further variation in the PTA of the assessed regimens as skin exposure to rifampicin may be lower than plasma.

References:

1. Shukla C, Patel V, Juluru R, Stagni G. Quantification and prediction of skin pharmacokinetics of amoxicillin and cefuroxime. Biopharm Drug Disp. 2009; 30(6):281-293.

2. Adam D, de Visser I, Koeppe P. Pharmacokinetics of amoxicillin and clavulanic acid administered alone and in combination. Antimicrob Agent Chemother. 1982; 22(3):353-7.

3 Gujral RS, Haque SM. Simultaneous determination of potassium clavulanate and amoxicillin trihydrate in bulk, pharmaceutical formulations and in human urine samples by UV spectrophotometry. Int J Biomed Sci. 2010; 6(4):335-43.

4. Shimomura H, Andachi S, Aono T, Kigure A, Yamamoto Y, Miyajima A, Hirota T, Imanaka K, Majima T, Masuyama H, Tatsumi K, Aoyama T. Serum concentrations of clarithromycin and rifampicin in pulmonary *Mycobacterium avium* complex disease: long-term changes due to drug interactions and their association with clinical outcomes. J Pharm Health Care Sci. 2015;1(1): 32.

5. Alffenaar JWC, Nienhuis WA, de Velde F, Zuur AT, Wessels AMA, Almeida D, Grosset J, Adjei O, Uges DRA, van der Werf TS. Pharmacokinetics of rifampin and clarithromycin in patients treated for *Mycobacterium ulcerans* infection. Antimicrob Agent Chemother 2010; 54(9):3878–3883.

6. Lyons MA, Reisfeld B, Yang RSH, Lenaerts AJ. A physiologically-based pharmacokinetic model of rifampin in mice. Antimicrobial Agents Chemother 2013; 57(4):1763-1771.

7. Kobuchi S, Kabata T, Maeda K, Ito Y, Sakaeda T. Pharmacokinetics of macrolide antibiotics and transport into the interstitial fluid: Comparison among erythromycin, clarithromycin, and azithromycin. Antibiotics 2020; 9(4); 199.
